# The Goal of Adequate Data

**Published:** 2008-06-15

**Authors:** Edward J Sondik

**Affiliations:** National Center for Health Statistics, Coordinating Center for Health Information and Services, Centers for Disease Control and Prevention

*"Timely and reliable data are an essential component of public health assessment, policy development, and assurance at all levels of government."*

— Institute of Medicine, *The Future of the Public's Health*

Responsible decision making requires adequate data — and the Institute of Medicine (IOM) report is one of several reports and initiatives that look forward to a new era of timely, high-quality information on health brought about by an improved data infrastructure in public health. Although we have excellent sources of information at the national level in the National Health Interview Survey and the National Health and Nutrition Examination Survey, and at the state and county levels in the Behavioral Risk Factor Surveillance System, each source has inherent limitations. More serious, however, are the issues raised by the 2002 report from the National Committee on Vital and Health Statistics (NCVHS) (1). That report emphasized the need for sufficient data to address the health needs of minority groups and, in particular, to account for various influences on our population's health. Among these influences are community attributes, including the economy and aspects of the built environment; contextual variables from the natural environment to the political context; social determinants of health; and influences of place and time. The NCVHS analysis noted an "unevenness in the [data] enterprise's capacity to provide data on all influences on population health" and identified a "shortage of state and local data, especially survey data" (1, p. ix) as one of the causes. Another recommendation for strengthening the information base for public health is establishment of a "uniform national data set . . . that will permit valid comparison of local and state health data with those of the nation and of other states and localities and that will facilitate progress toward national health objectives and implementation" (2).

The Community Health Status Indicators (CHSI) project (3) is a sign of such progress, bringing together data from multiple sources and multiple domains to aid in county-level decision making. Like the CHSI project, other efforts on the horizon could change our data sources and meet the needs outlined by the NCVHS and the IOM. For example:

*Community indicators.* The National Academy of Sciences and other organizations are involved in developing a set of key national indicators for the United States. These indicators would include health as one of their dimensions. Although this project is national, similar efforts are under way in U.S. communities, literally from coast to coast (for example, the Santa Cruz County Community Assessment Project in California and the CitiStat in Baltimore, Maryland). The rapid growth of indicators or measures that inform the public and professionals alike in an easily accessible form is encouraging.

*Electronic health records. *The use of electronic health records received a major boost from President George W. Bush and Secretary of Health and Human Services Michael Leavitt with establishment of the Office of the National Coordinator for Health Information Technology. Public health information is one of the foci of this organization and others, including the American Health Information Community (AHIC), a federally chartered advisory committee on the use and adoption of electronic health records. To assess the potential impact of electronic health records on health statistics and on public health decision making, the office of the Assistant Secretary for Health and the National Center for Health Statistics sponsored a workshop, and the report is available online (www.cdc.gov/nchs/data/nhcs/EMRworkshopsummaryjuly30.pdf).

Healthy People. Perhaps the leading force in developing new data sources and emphasizing the importance of standardization in definitions and methods is the Health and Human Services *Healthy People* program, now in its third decade. Although the *Healthy People* focus is national, it places a strong emphasis on developing data for local use. A recent paper shows that this goal is indeed feasible with a strong research base (4). 

Although these developments herald progress, critical gaps remain in our knowledge and data. As the nation grows more diverse in race, ethnicity, and various other factors, the need for adequate data to address health problems will multiply with each distinct population group. Furthermore, our definition of health must be broadened so that functioning is included as a part of our health assessment.

Unfortunately, the current supporting resource base needs to be strengthened. CDC Director Julie Gerberding noted in CDC's Professional Judgment Budget Request that "CDC's mission-critical health statistics and similar data systems are currently on life support. Investments have simply not kept pace with expenses and technological advances." As we work to achieve the promise of new sources and new tools, we also need to ensure that we do not damage the critical national data infrastructure. Our investments in standards development must be balanced with investments in tools, training, analysis, and research. 

Despite these constraints on resources, we continue to make progress in collecting and disseminating information crucial for public health decisions. The year 2007 marked the 50th anniversary of the National Health Interview Survey, an appropriate time to stress the gains we have made in health data and a commitment to working toward a future of relevant and high-quality information at every level of health decision making.
